# What matters to patients and clinicians when discussing the impact of cancer medicines on health-related quality of life? Consensus-based mixed methods approach in prostate cancer

**DOI:** 10.1007/s00520-021-06724-6

**Published:** 2021-12-08

**Authors:** Emma Dunlop, Aimee Ferguson, Tanja Mueller, Kelly Baillie, Julie Clarke, Jennifer Laskey, Amanj Kurdi, Olivia Wu, Rob Jones, Hilary Glen, Marion Bennie

**Affiliations:** 1 Strathclyde Institute of Pharmacy & Biomedical Sciences (SIPBS), University of Strathclyde, Glasgow, UK; 2 NHS Greater Glasgow & Clyde, Glasgow, UK; 3 Department of Pharmacology, College of Pharmacy, Hawler Medical University, Erbil, Iraq; 4 HEHTA Research Unit, University of Glasgow, Glasgow, UK; 5 Institute of Cancer Sciences, University of Glasgow, Beatson West of Scotland Cancer Centre, 1053 Great Western Road, Glasgow, G12 0YN UK; 6 Beatson West of Scotland Cancer Care, 1053 Great Western Road, Glasgow, G12 0YN UK

**Keywords:** Patient-reported outcomes, PROMs, Prostate cancer, Health-related quality of life, Mixed methods, Consensus methods

## Abstract

**Objective:**

To identify what matters to clinicians and patients when discussing cancer medicines’ impact on health-related quality of life (HRQoL).

**Methods:**

A framework of HRQoL domain/domain elements was developed, informed by analysis of published patient reported outcome measures (PROMs), applicable to prostate cancer. Using mixed methods (eDelphi, Nominal Group Technique and questionnaire), prostate cancer clinicians and patients attending prostate cancer clinics and support groups were asked which domains/domain elements would be important to them when discussing the impact prostate cancer medicines have on their HRQoL.

**Results:**

Twenty-one clinicians and 71 patients participated from the West of Scotland. Clinicians and patients identified 53/62 domain elements across seven domains as important, of which 32 (60%) were common to both groups. Clinicians placed more importance than patients on *Mood & Emotion*; in contrast, patients placed importance on a broader range of *Symptoms & Side Effects,* being informed about their care, and having effective healthcare professional collaboration.

**Conclusion:**

This study provides insight into the similarities and differences between what clinicians and patients think is important when discussing the impact of cancer medicines on HRQoL. Future research should involve exploring the potential for consistency of medicines PROMs across different cancer types to support patient-clinician communication and drive improvements in care.

**Supplementary Information:**

The online version contains supplementary material available at 10.1007/s00520-021-06724-6.

## Introduction

Cancer is the second leading cause of death globally [1]. Prostate cancer is the most common cancer among men in Europe, accounting for 24% of all newly diagnosed cancers in 2018 with an estimated 450,000 new diagnoses [2]. While incidence is rising — partly based on more widespread access to testing — survival across Europe has increased [3], mainly due to an increased availability of systemic anticancer therapies (SACT) [4, 5].

As survival rates improve and more patients are living with cancer, a shift away from solely offering supportive palliative care at the end of life [5] has resulted in an interest in assessing Quality of Life (QoL) among cancer patients throughout their journey [6], in addition to standard clinical outcomes such as overall survival. Measurement of health-related quality of life (HRQoL), referring to the impact of health, illness, and treatment on QoL [7], is recognised as important at both the patient and population level. At a patient level, the ability to monitor treatment impact on a patient’s well-being can support patient/clinician decision-making about treatment and care, and may improve outcomes [8]; at the population level, HRQoL is increasingly being considered as part of the assessment of overall clinical effectiveness and the value of cancer medicines [9, 10].

Although assessing patients’ experiences of treatment is a central component of healthcare and, in 2005, Ferrans et al. proposed a model for measuring HRQoL that comprised elements of biological function, symptoms, functionality, general health perceptions, and overall quality of life [7], there is a lack of agreement regarding the method of measuring HRQoL, or what elements should indeed be included as part of a full package of patient care [11–17]. Research within the field focuses on the use of patient reported outcome measures (PROMs) — a common means of measuring and comparing HRQoL. While PROM tools are frequently and successfully used in clinical trials [18, 19], widespread application of PROMs is uncommon in routine clinical practice. Key barriers regarding their routine use in clinical practice range from misconceptions about the value of PROMs to technical difficulties with integrating suitable tools into clinical workflows, a prerequisite for implementation at scale; furthermore, successful implementation requires frequent and consistent interaction with both healthcare staff as well as patients and their carers, since engaging stakeholders and sustaining the completion of PROMs tools can be challenging [20]. These problems are compounded by the large number of different PROMs tools already available, and the differences in their relevance and applicability depending on context. As the relevance of PROM tools is a factor not only affecting their potential usefulness for clinicians but also the retention of patients engaging with them, the variability of tools available — and the wide range of specific topics covered — poses questions of suitability of individual tools, with implications for future decisions about which tool(s) to choose going forward [10, 21, 22].

Nevertheless, regular collection of PROMs could offer clinicians a more systematically assessed view of how patients are tolerating treatment; support discussions with patients; and facilitate more informed collaborative decision-making with regard to their cancer treatment [8, 21, 23, 24]. Engaging patients, clinicians, other healthcare staff, academics, and policy makers in the design and development of HRQoL PROMs strategies ensures that they meet stakeholder needs and focus on the importance, relevance, and completeness of content of PROMs tools used to support care [21, 25].

The continually evolving prostate cancer treatment landscape comprises a broad range of cancer medicines, which are appropriate for use at different stages of disease [26, 27]. PROMs research in prostate cancer has focused mainly on the impact of the disease on HRQoL and on measuring acute toxicity from systematic anticancer treatments (SACT) [28–30]***. ***Less is known about the impact of cancer medicines more generally on a patient’s wellbeing and their supportive care needs.

In line with the Scottish National Cancer Strategy [31], the Cancer Medicines Outcomes Programme (CMOP) is funded by the Scottish Government to test the feasibility of collecting HRQoL data from clinical practice to support clinical decision-making, with a focus on understanding what PROMs data could be collected and utilised as part of routine care, and how best to accomplish this. The aim of this study was to identify what matters to prostate cancer patients and clinicians when discussing the impact of cancer medicines on HRQoL to better understand the care needs of these patients, and inform future PROMs data collection.

## Methods

There is an extensive body of work regarding PROMs tools in cancer. However, existing tools being used to assess HRQoL vary in the elements they aim to capture; how well their development is evidenced (including reliability and validity testing); and their overall usefulness. As the landscape of PROMs tools is already vast, it was deemed less useful to design a new PROMs tool for use in cancer medicines. Instead, based on a literature review aimed at identifying available PROMs tools to inform the study material, consensus methods were used to establish which areas of HRQoL represented within existing tools are most important to the study population, with a view to exploring what is valuable in the tools already available. Consensus methods are applied extensively in healthcare research [32–34]; a mixed methods approach was adopted in order to accommodate the specific needs of patients and clinicians.

## Study material development

PROMs tools relevant for use in a prostate cancer patient population (including generic QoL, health, cancer, and prostate cancer-specific tools) available in English and either reliability and validity tested or endorsed by healthcare organisations were searched for in three databases (PubMed, Science Direct, and Google Scholar). The search terms used were as follows: “PROMs” and its full forms (“patient reported outcome measures”, “patient reported outcomes”); “prostate cancer”; “Quality of Life” and the acronyms “QoL” and “HRQoL”; and “qualitative”. Additional information regarding suitable PROMs tools were provided by members of the research team with experience in cancer PROMs. Literature searches were conducted between December 2016 and May 2017 until no further PROMs tools could be identified, resulting in a total of 30 potentially relevant tools. These were subsequently discussed by the CMOP team — comprising academics as well as clinicians — to ensure relevance to clinical practice; all identified tools were confirmed as relevant.

**Fig. 1:**
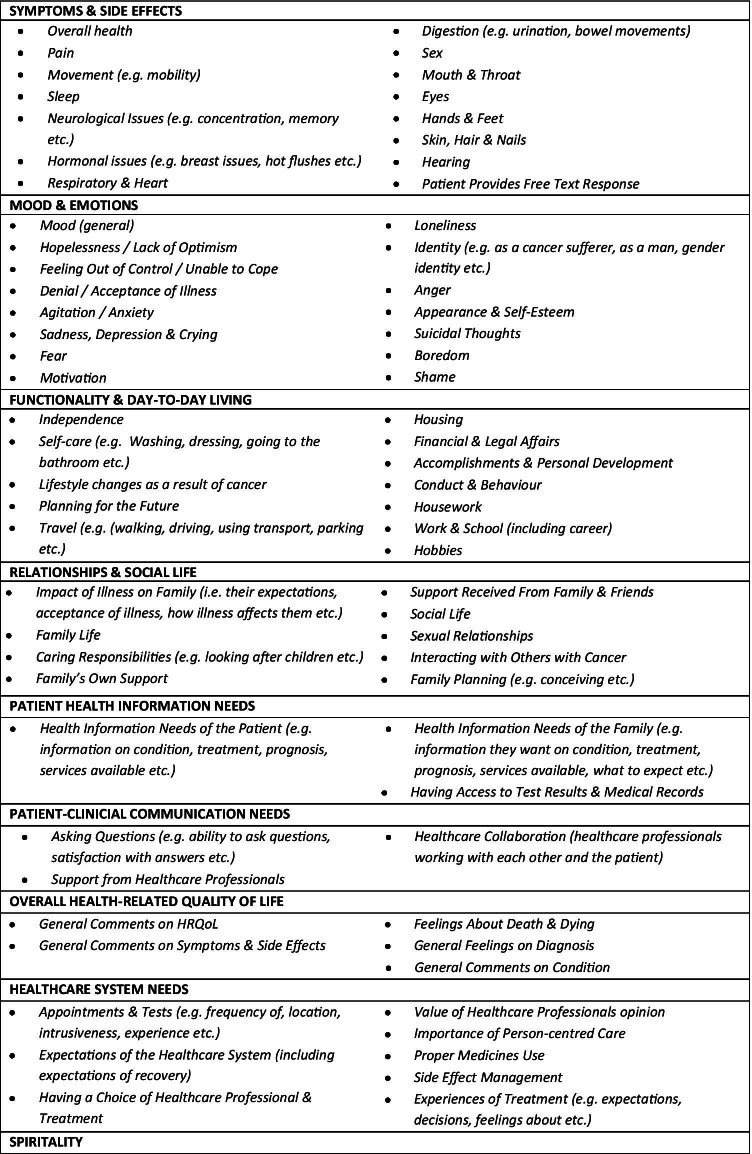
List of HRQoL domains (*n* = 9) and domain elements (*n* = 70) derived from selected PROMs tools (*n* = 30)

Using NVivo v11, the 30 identified PROMs tools were coded by one researcher in terms of what each question within these tools addressed. After validation by a second researcher, the coding was used to generate a framework of domains and domain elements; this framework was then validated against published theories and definitions of QoL and HRQoL by conducting a matching exercise, ensuring that all vital elements were captured [7, 35–37]. The developed framework, comprising nine domains and 70 domain elements (Fig. 1), was subsequently used to generate the data collection tools used throughout this study (eDelphi questionnaires, NGT workbooks, and Clinic Questionnaires).

## Identification of study participants

Clinicians — including oncologists, urologists, pharmacists, nurses, dieticians, and physiotherapists — engaged in prostate cancer clinics in hospitals within the West of Scotland Cancer Network (WoSCAN) were eligible to participate and were identified through the CMOP clinical network.

Patients eligible for participation were identified in two ways:

- Attendance of either one of two prostate cancer support groups hosted in NHS Greater Glasgow & Clyde (NHS GGC); support groups were identified via internet search.
- Attendance at prostate cancer clinics in two NHS GGC hospitals, and currently or previously on prescribed medicines for prostate cancer treatment; clinics were identified via the CMOP clinical network.

## Data collection and analyses

### Clinicians

Clinicians were invited via email to participate in an eDelphi, consisting of two rounds and taking place between October and December 2017. All potential participants were provided with electronic participant information sheets (PIS) and consent forms to complete prior to taking part.

The first eDelphi questionnaire asked clinicians to rank the importance of the nine framework domains (Fig. 1) in relation to discussing with patients the impact of medicines on their HRQoL (1 — least important/relevant; to 9 —most important/relevant). Responses were analysed by summing the rank scores and calculating the median/interquartile range (IQR) for each domain; domains with median scores on or above the pre-defined threshold (the mid-point of the scale, i.e. 5) were retained. Domains with median scores below the threshold but with a wide IQR encompassing the threshold were reviewed by the CMOP team [38].

The second eDelphi questionnaire presented the retained domains back to clinicians and asked them to rank each of the domain’s elements from least to most important/relevant. Responses were analysed as per eDelphi 1; domain elements with median scores on or above the pre-defined threshold (the mid-point of the scale, which varied depending on the number of domain elements per domain) were retained, while those below the threshold but with a wide IQR encompassing the threshold were reviewed by the CMOP team.

### Patients

Researchers visited prostate cancer support groups on several occasions between February and July 2018. On these days, patients who were present were invited to participate in a group consensus approach called Nominal Group Technique (NGT) [39], and were provided with PIS and consent forms to complete prior to commencement of group activities.

**Fig. 2:**
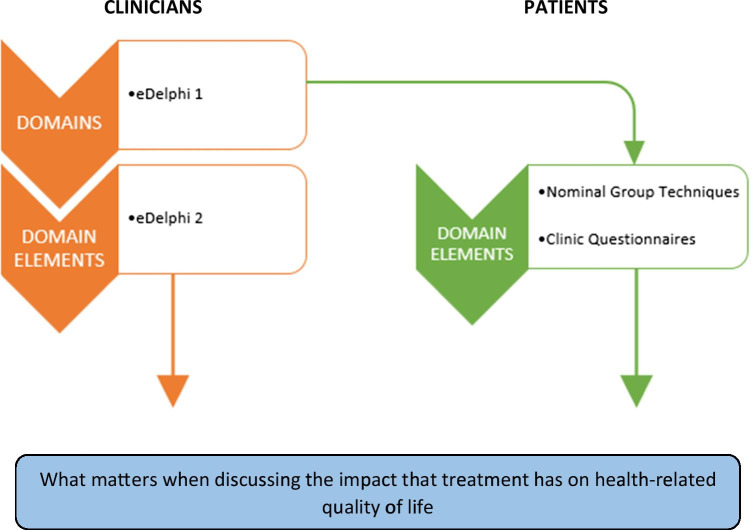
Data collection methods applied for clinicians and patients

Each NGT focused on a different domain and its associated elements, and lasted 1–1.5 h. Participants were led through a structured process to consider the importance of the domain elements previously identified through eDelphi 1 in relation to the impact of medicines on their HRQoL (Fig. 2). The process involved participants writing their thoughts on each domain element in the NGT workbook; verbalising these thoughts to the group one by one; an open group discussion; individual scoring of each domain element using a Likert scale (1 — not important at all; to 5 — extremely important); collation of scores to generate a ranked list of the domain elements by the researcher; and presenting the established list back to the group for discussion, followed by anonymised voting to indicate group consensus.

Additionally, patients attending prostate cancer clinics between June and August 2018 were recruited by the consulting clinicians, who introduced the study to eligible patients. If interested, patients were referred to the researcher’s present in-clinic; the researchers explained the implications of participation and provided PIS and consent forms. Participants then received the Clinic Questionnaire (a revised version of the NGT workbook comprising all domains and domain element as identified through eDelphi 1; Fig. 2). They were informed that completed questionnaires could be deposited into a sealed box placed at the clinic reception; they also received a pre-stamped return envelope in case they preferred completing the questionnaire at home.

Individual patients’ Likert scores from the NGTs and the Clinic Questionnaires were collated and analysed by calculating medians; domain elements with a median Likert score above the pre-defined threshold (the mid-point of the scale, i.e. 3) were retained. The qualitative data from NGT discussions and free text comments in the questionnaires were transcribed, validated, and analysed using NVivo v11 to capture the patients’ voice and provide context as to why specific domain elements were important to the patient population [40].

## Results

### Participants

Of 146 invited clinicians, 21 participated in eDelphi 1, all of whom progressed to complete eDelphi 2. Clinicians represented various roles (8 oncologists, 4 urologists, 5 nurses, and 4 pharmacists), with experience in their current role ranging from < 1 to 17 years; mean [SD] age was 46.5 [9.6] years, and 57.1% (*n* = 12) were female.

Thirty unique patients participated in the NGTs; the mean [SD] age was 68.8 [6.6] years, and patients had lived with prostate cancer for a median of 2.75 [IQR 1–9] years. The Clinic Questionnaire was completed by 41 patients with a mean [SD] age of 73.8 [7.5] years.

### Framework domains

Six out of the original nine domains received a median rank score above the pre-defined threshold in eDelphi 1, and three domains had a median score below the threshold but with a wide IQR containing the threshold. While the former were retained, two of the latter were removed following review by the CMOP team (“Patients experience of the Healthcare System” and “Spirituality”). The resulting framework therefore comprised seven domains, with a total of 62 domain elements (between 3 and 15 elements in each domain).

### Framework domain elements

**Fig. 3:**
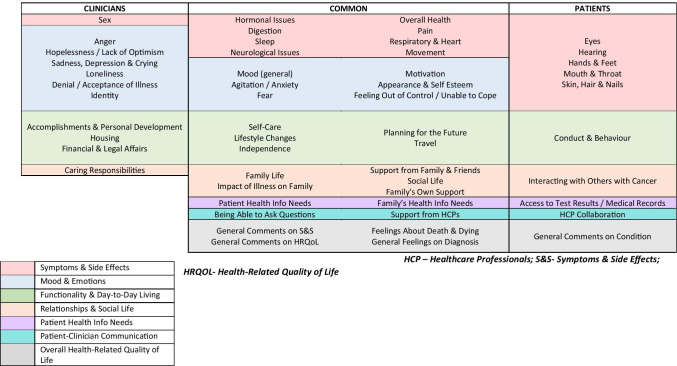
Health-related quality of life domain elements (*n* = 53) important to clinicians and patients

Following analysis and review of responses obtained through eDelphi 2, 43 domain elements were identified as being important to clinicians; based on analyses of responses from NGTs and Clinic Questionnaires, 42 domain elements were identified as being important to patients. Figure 3 illustrates that 32 domain elements across the seven domains were important to both clinicians and patients, with a further 11 and 10 specific to clinicians and patients, respectively; while clinicians placed more importance on certain elements of “Mood & Emotion”, patients highlighted additional aspects of “Symptoms & Side Effects”, as well as elements around their information and communication needs. Further details can be found in Supplementary file 1.

Comparing the results obtained through NGTs and Clinic Questionnaires showed that clinic patients were most concerned with issues relating to symptoms and side effects, whereas support group patients focused on aspects of living with cancer and mental health impacts (Supplementary file 2).

### Patient voice

The patient voice, which provides context to the physical, emotional, and lifestyle impact of cancer medicines treatment on HRQoL, is represented in Table 1; captured from NGTs and Clinic Questionnaires, quotes are aligned with the domains and domain elements of the developed framework.

**Table 1:** Patient voice — illustrative quotes aligned to the domains/domain elements

Domain	Illustrative quote
Symptoms & Side Effects	“…you’re tossing and turning with pain…You’ll be lying on your side and you’re sore on that side, so you go on the other side, then you get sore on that one” (NGT, 61yrs, domain elements—Sleep and Pain)
Mood & Emotion	“I have to say, however necessary it is in all range of illnesses, to go to hospital, it’s like taking your car to Kwikfit [a mechanic], they don’t wanna know about the emotions, they don’t wanna know the very thing that makes us human…And when you know that the trajectory of your illness is related to your emotions, the whole person been brought to a thing, that is the genius of [the support group]. And when these two meet, we will have a medicine, we will have an outcome that I think will alter the trajectory of all illnesses, not just cancer. (NGT, 58yrs, domain element Sadness, Depression & Crying)
Functionality & Day-to-Day Living	“Coping with the number of pills/capsules, eye drops and nasal spray presents problems. The incessant routine of having to check supply of the 20 items that I have been prescribed is a burden.” (Clinic Questionnaire, 84yrs, domain element – Lifestyle Changes)
Relationships & Social Life	“Sometimes you don’t feel like going out…I don’t mean I’m antisocial or anything…But it’s all down to the hormone treatment, I think.” (NGT, 61yrs, domain element—Social Life)
Patient Information Needs	“It’s very important to receive [health] information, probably more for the family” (NGT, 68yrs, domain element – Your Family’s Health Information Needs)
Patient-Clinician Communication Needs	“I have the ability to ask questions. What is important is to have freedom to ask, or being invited to do so. And the freedom to express the degree of satisfaction/dissatisfaction with the answers” (Clinic Questionnaire, 84yrs, domain element – Being Able to Ask Questions)
Overall QoL	“A lot of people would trade a couple of extra months of the chemo, maybe a year, maybe a few months less comfortably, so [addressing the impact treatment has on overall Quality of Life] is an option, an important one” (NGT, 80yrs, domain element—General Comments on QoL)

## Discussion

### Key findings

The purpose of this study was to identify what matters to prostate cancer patients and clinicians when discussing cancer medicines’ impact on HRQoL. From an analysis of published PROMs tools applicable to prostate cancer, a HRQoL framework composed of nine domains and 70 domain elements was created. Clinicians and patients identified a total of 53 domain elements across seven domains as important when discussing the impact of their cancer treatment, of which 32 (60%) were common to both groups. Interestingly, clinicians placed more importance than patients on “Mood & Emotion”; in contrast, patients placed more importance on a broader range of “Symptoms & Side Effects”, and prioritised being informed about their care and having effective collaboration across healthcare professionals.

Previous literature has focused on the level of agreement or concordance between clinicians and patients in their assessment of HRQoL, including during active cancer treatment (principally SACT) and in palliative care, and agreement is commonly greater when examining physical, objective symptoms (e.g. vomiting, diarrhoea) rather than subjective/psychological symptoms (e.g. fatigue, anxiety) [11–17, 41]. In our study, we had good agreement between clinicians and patients across a range of both physical (“Symptoms and Side Effects”, “Functionality & Day to Day Living”) and psychological (“Mood & Emotion”) domains (Fig. 3). Notably, patients identified a broader range of additional “Symptoms & Side Effects” elements as important. This difference might in part be attributable to clinicians underestimating the severity of physical symptoms, as highlighted in other studies [14, 22]. In contrast, clinicians identified a wider range of “Mood & Emotions” elements as important; although patients signalled their need for broad support (e.g. including “Interacting with Others with Cancer”), they may not necessarily expect this solely to be accessed through their clinician, but through signposting to other systems such as support groups.

Interestingly, we observed that patients who completed clinic questionnaires placed greater importance on a wider range of symptoms and side effects in comparison to those who participated in the support group NGTs, whereas patients recruited from support groups also prioritised elements from the following domains: “Functionality & Day to Day Living”; “Relationships and Social Life”; and “Mood and Emotion” (supplementary file 2). This variation in prioritisation of HRQoL domains is in agreement with the wider evidence base that shows patients may have discrete information needs across their cancer journey, influenced by multiple factors including the different treatments available to patients throughout their illness; the life-limiting side effects these treatments can bring; and how they impact on a patient’s support and care needs [42, 43]. Clinicians need to be cognisant of patients’ diverse and changing areas of importance, and more systematic gathering and exchange of information across the patient journey could better support dialogues as part of shared decision-making and supportive care.

### Strengths and limitations

To our knowledge, this is the first study to examine specifically clinicians’ and patients’ perspectives of what areas of HRQoL matter and are important to understanding the impact of cancer medicines, rather than the level of agreement or concordance at a point in time when independently assessing HRQoL. We adopted a mixed methods approach, advocated as good practice in health outcomes research [44]. The review of the literature to develop a framework of domains exploited established knowledge and formed the basis of the study materials; we also provided the participants the opportunity to contribute any additional domain/domain elements that they felt were important but were not represented. We did not ask participants to generate their own domains and domain elements, which may be perceived as a study limitation. However, we perceived this as too cognitively demanding, and it may have resulted in a brief, limited list, while not benefitting from the existing evidence base.

We chose to engage with patients from two diverse settings (i.e. patient support group in a non-healthcare setting and a hospital clinic) to understand better any influence on considering HRQoL. Although this required two different methods of data collection in these divergent environments (NGT and clinic questionnaires), both methods contained the same domain/domain elements. NGT participants may have been influenced by their peers’ opinions in the discussion prior to scoring; nevertheless, participants scored each domain element individually, and these data were used in the quantitative analysis (Fig. 3, Supplementary file 1). The NGT also enabled the capture of group discussions, thereby adding a helpful patient voice to the quantitative data interpretation (Table 1).

We did not document where the patients were on their cancer pathway, only that they had received at least one cancer medicine, and acknowledge that this may impact the generalisability of our findings. We also recognise the potential bias in sampling as patients may attend support groups for specific emotional or information-based needs appropriate for this setting, perhaps related to having negative or less favourable experiences of treatment.

Similarly, the overall number of clinicians participating in the first Delphi phase of this project was limited, and participating clinicians may not be a representative sample; this is of particular relevance as results from this first phase informed all subsequent stages. Nevertheless, we do not expect this to have a substantial impact on our findings since participants represented a broad spectrum of experiences, both in terms of profession and years of clinical practice, and it has been acknowledged that the composition of Delphi panels — and its combined expertise in relation to the topic in question — is more important than its size in order to elicit meaningful responses [45].

### Future directions for PROMs use in clinical practice

Within the evolving evidence base, there is a rapidly growing interest in how PROMs can be streamlined into routine clinical practice to provide best supportive care. Recent publications discuss the potential benefits of broad PROMs adoption within healthcare systems to support better communication between patients and clinicians and inform improvements in service provision; this is in line with our study findings suggesting that more consistent gathering and exchange of information across the patient journey could support dialogues between patients and their clinicians as part of shared decision-making and supportive care. Ideally, going forward, dynamic PROMs tools — modifiable in order to be adaptive to changing circumstances — should be integrated into routine clinical practice to enable this. For example, this could involve the consistent use of PROMs tools that have questions relevant to the impacts of specific treatments on HRQoL, alongside other PROMs tools depending on the patient’s priorities or care needs.

Future research should consider how findings of this study can support the use and/or adaptation of established PROMs, i.e. how fit for purpose and complete present PROMs tools are to meet identified needs. A programme of work to assess and adopt/adapt PROMs collection and utilisation through co-design with stakeholders (and integration within cancer care pathways) should be progressed. Moving forward, thought should also be given to the potential for consistency of medicines PROMs across different cancer types, with cancer medicines often being used across multiple cancers, to support and inform a national cancer medicines PROMs strategy. Such a strategy could better enable the collection and use of PROMs data as part of routine care, support treatment regimes, aid system integration, improve patient care, and facilitate public health impact evaluation.

In line with the vision in Scotland for a strategic co-ordinated approach to the adoption of PROMs [31, 46], our ongoing work involves matching the study output domains and domain elements prioritised by patients and clinicians to recognised, validated PROMs tools; and co-designing a digital prototype for patients to input PROMs and a clinical dashboard integrated within the clinical health information technology system. Our ambition is to explore further the potential level of standardisation of PROMs in content, layout, and appearance to minimise burden on patients through their cancer journey, and for clinicians as they manage patients across multiple diseases and treatments. This ambition is shared with colleagues nationally and internationally, calling for a more efficient co-ordinated approach to the adoption and assessment of PROMs within health systems if we are to realise the benefits for patients and society [10, 21].

## Conclusion

This study provides insight into the similarities and differences between what prostate cancer clinicians and patients wish to discuss regarding the impact cancer medicines have on HRQoL. There was good consensus between clinicians and patients but also some interesting differences were identified, which warrant further research. Such research should also consider exploring the potential for consistency of medicines PROMs across different cancer types to support patient-clinician communication and drive improvements in supportive care.

## Supplementary Information

Below is the link to the electronic supplementary material.

Supplementary file1 (DOCX 19 kb)

Supplementary file2 (DOCX 17 kb)

## Data Availability

There is no data available.

## Code availability

NA.
