# Supplementary material for: What matters to patients and clinicians when discussing the impact of cancer medicines on health-related quality of life? Consensus-based mixed methods approach in prostate cancer
Source: Support Care Cancer. 2021 Dec 8;30(4):3141–50. doi: 10.1007/s00520-021-06724-6 (PMC8857102; doi:10.1007/s00520-021-06724-6)
Supplement: Supplementary file 1 — Supplementary file1 (DOCX 19 kb) [file 520_2021_6724_MOESM1_ESM.docx]

**What matters to patients and clinicians when discussing the impact of cancer medicines on health-related quality of life?**  **Consensus-based mixed methods approach in prostate cancer**

**Journal: Supportive Care in Cancer**

Ms Emma Dunlop, Strathclyde Institute of Pharmacy & Biomedical Sciences (SIPBS), University of Strathclyde, Glasgow, UK

Miss Aimee Ferguson, Strathclyde Institute of Pharmacy & Biomedical Sciences (SIPBS), University of Strathclyde, Glasgow, UK

Dr Tanja Mueller, Strathclyde Institute of Pharmacy & Biomedical Sciences (SIPBS), University of Strathclyde, Glasgow, UK

Mrs Kelly Baillie, NHS Greater Glasgow & Clyde, Glasgow UK

Ms Julie Clarke, NHS Greater Glasgow & Clyde, Glasgow UK

Mrs Jennifer Laskey, NHS Greater Glasgow & Clyde, Glasgow UK

Dr Amanj Kurdi, Strathclyde Institute of Pharmacy & Biomedical Sciences (SIPBS), University of Strathclyde, Glasgow, UK; Department of Pharmacology, College of Pharmacy, Hawler Medical University, Erbil, Iraq

Prof Olivia Wu, HEHTA Research Unit, University of Glasgow, Glasgow, UK

Dr Rob Jones, PhD MBChB, Institute of Cancer Sciences, University of Glasgow, Beatson West of Scotland Cancer Centre, 1053 Great Western Road, Glasgow G12 0YN

Dr Hilary Glen, Beatson West of Scotland Cancer Care, 1053 Great Western Road, Glasgow, G12 0YN

Prof Marion Bennie, Strathclyde Institute of Pharmacy & Biomedical Sciences (SIPBS), University of Strathclyde, Glasgow, UK

**Corresponding Author:** Miss Aimee Ferguson / a.ferguson@strath.ac.uk

**Supplementary File 1**

**Supplementary 1.** The domain elements important to clinicians and patients

| Domain Elements (n= 62) | **Clinician Data** | | **Patients Data** | |
| --- | --- | --- | --- | --- |
| **SYMPTOMS & SIDE EFFECTS** | Important  (Predefined Threshold = 8, range 1-15) † | Median | Important  (Predefined Threshold = 3  range 1-5) ‡ | Median |
| Overall Health | ✓ | 15 | ✓ | 4 |
| Pain | ✓ | 14 | ✓ | 4 |
| Movement | ✓ | 12 | ✓ | 4 |
| Sleep | ✓ | 11 | ✓ | 4 |
| Neurological Issues | ✓ | 10 | ✓ | 4 |
| Hormonal Issues | ✓ | 10 | ✓ | 4 |
| Respiratory & Heart | ✓ | 9 | ✓ | 4 |
| Digestion | ✓ | 8 | ✓ | 4 |
| Sex | ✓ | 7 § |  | 2 |
| Mouth & Throat |  | 6 | ✓ | 4 |
| Eyes |  | 5 | ✓ | 4 |
| Hands & Feet |  | 5 | ✓ | 4 |
| Skin, Hair & Nails |  | 5 | ✓ | 4 |
| Hearing |  | 4 | ✓ | 4 |
| **MOOD & EMOTION** | Important  (Predefined Threshold = 8, range 1-15) † | Median | Important  (Predefined Threshold = 3  range 1-5) ‡ | Median |
| Mood (general) | ✓ | 13 | ✓ | 4 |
| Hopelessness / Lack of Optimism | ✓ | 11 |  | 3 |
| Feeling Out of Control / Unable to Cope | ✓ | 10 | ✓ | 4 |
| Denial / Acceptance of Illness | ✓ | 10 |  | 3 |
| Agitation / Anxiety | ✓ | 10 | ✓ | 4 |
| Sadness, Depression & Crying | ✓ | 10 |  | 3 |
| Fear | ✓ | 9 | ✓ | 4 |
| Motivation | ✓ | 9 | ✓ | 4 |
| Loneliness | ✓ | 8 |  | 3 |
| Identity | ✓ | 7 § |  | 3 |
| Anger | ✓ | 7 § |  | 3 |
| Appearance & Self-Esteem | ✓ | 6 § | ✓ | 4 |
| **FUNCTIONALITY & DAY TO DAY LIVING** | Important  (Predefined Threshold = 6.5  range 1-12) † | Median | Important  (Predefined Threshold = 3  range 1-5) ‡ | Median |
| Independence | ✓ | 11 | ✓ | 4 |
| Self-care | ✓ | 10 | ✓ | 4 |
| Lifestyle Changes as a Result of Cancer | ✓ | 8 | ✓ | 4 |
| Planning for the Future | ✓ | 7 | ✓ | 4 |
| Travel | ✓ | 7 | ✓ | 4 |
| Housing | ✓ | 5 § |  | 1 |
| Financial & Legal Affairs | ✓ | 5 § |  | 2 |
| Accomplishments & Personal Development | ✓ | 4 § |  | 3 |
| Conduct & Behaviour |  | 4 | ✓ | 4 |
| **RELATIONSHIPS & SOCIAL LIFE** | Important  (Predefined Threshold = 5  range 1-9) † | Median | Important  (Predefined Threshold = 3  range 1-5) ‡ | Median |
| Impact of Illness on Family | ✓ | 8 | ✓ | 4 |
| Family Life | ✓ | 7 | ✓ | 4 |
| Caring Responsibilities | ✓ | 7 |  | 3 |
| Family's Own Support | ✓ | 6 | ✓ | 4.5 |
| Support from Family & Friends | ✓ | 6 | ✓ | 4.5 |
| Social Life | ✓ | 4 § | ✓ | 4 |
| Interacting with Others with Cancer |  | 2 | ✓ | 3.5 |
| **PATIENT HEALTH INFORMATION NEEDS** | Important  (Predefined Threshold = 2  range 1-3) † | Median | Important  (Predefined Threshold = 3  range 1-5) ‡ | Median |
| Health Information Needs of Patient | ✓ | 3 | ✓ | 5 |
| Health Information Needs for the Family | ✓ | 2 | ✓ | 4 |
| Having Access to Test Results & Medical Records |  | 1 | ✓ | 4 |
| **PATIENT-CLINICIAN COMMUNICATION NEEDS** | Important  (Predefined Threshold = 2  range 1-3) † | Median | Important  (Predefined Threshold = 3  range 1-5) ‡ | Median |
| Being Able to Ask Questions | ✓ | 3 | ✓ | 5 |
| Support from Healthcare Professionals | ✓ | 2 | ✓ | 4 |
| Healthcare Professional Collaboration |  | 1 | ✓ | 4 |
| **OVERALL QOL** | Important  (Predefined Threshold = 3  range 1-5) † | Median | Important  (Predefined Threshold = 3  range 1-5) ‡ | Median |
| General Comments on QoL | ✓ | 4 | ✓ | 4 |
| General Comments on Symptoms & Side Effects | ✓ | 4 | ✓ | 5 |
| Feelings About Death & Dying | ✓ | 3 | ✓ | 4 |
| General Feelings on Diagnosis | ✓ | 2 § | ✓ | 4 |
| General Comments on Condition |  | 2 | ✓ | 4 |
|  | **(n=43)** |  | **(n=42)** |  |

† Range differed depending on the number of domain elements per domain. All ranked from least important to most important

‡ Range: 1 – not at all important, 5 – extremely important

§ Retained due to large IQR following discussion and agreement by CMOP team (which included clinicians specialising in prostate cancer care)
