# Supplementary material for: What matters to patients and clinicians when discussing the impact of cancer medicines on health-related quality of life? Consensus-based mixed methods approach in prostate cancer
Source: Support Care Cancer. 2021 Dec 8;30(4):3141–50. doi: 10.1007/s00520-021-06724-6 (PMC8857102; doi:10.1007/s00520-021-06724-6)
Supplement: Supplementary file 2 — Supplementary file2 (DOCX 17 kb) [file 520_2021_6724_MOESM2_ESM.docx]

**What matters to patients and clinicians when discussing the impact of cancer medicines on health-related quality of life?**  **Consensus-based mixed methods approach in prostate cancer**

**Journal: Supportive Care in Cancer**

Ms Emma Dunlop, Strathclyde Institute of Pharmacy & Biomedical Sciences (SIPBS), University of Strathclyde, Glasgow, UK

Miss Aimee Ferguson, Strathclyde Institute of Pharmacy & Biomedical Sciences (SIPBS), University of Strathclyde, Glasgow, UK

Dr Tanja Mueller, Strathclyde Institute of Pharmacy & Biomedical Sciences (SIPBS), University of Strathclyde, Glasgow, UK

Mrs Kelly Baillie, NHS Greater Glasgow & Clyde, Glasgow UK

Ms Julie Clarke, NHS Greater Glasgow & Clyde, Glasgow UK

Mrs Jennifer Laskey, NHS Greater Glasgow & Clyde, Glasgow UK

Dr Amanj Kurdi, Strathclyde Institute of Pharmacy & Biomedical Sciences (SIPBS), University of Strathclyde, Glasgow, UK; Department of Pharmacology, College of Pharmacy, Hawler Medical University, Erbil, Iraq

Prof Olivia Wu, HEHTA Research Unit, University of Glasgow, Glasgow, UK

Dr Rob Jones, PhD MBChB, Institute of Cancer Sciences, University of Glasgow, Beatson West of Scotland Cancer Centre, 1053 Great Western Road, Glasgow G12 0YN

Dr Hilary Glen, Beatson West of Scotland Cancer Care, 1053 Great Western Road, Glasgow, G12 0YN

Prof Marion Bennie, Strathclyde Institute of Pharmacy & Biomedical Sciences (SIPBS), University of Strathclyde, Glasgow, UK

**Corresponding Author:** Miss Aimee Ferguson / a.ferguson@strath.ac.uk

**Supplementary File 2**

| **CLINIC PATIENTS** | **COMMON** | | **SUPPORT GROUP PATIENTS** |
| --- | --- | --- | --- |
| Eyes  Hearing  Hands & Feet  Mouth & Throat  Overall Health | Hormonal issues  Neurological Issues  Respiratory & Heart  Sleep | Movement  Pain  Digestion  Skin hair and nails | Sex |
|  |  |  | Anger  Sadness Depression & Crying  Denial / Acceptance of Illness  Fear  Hopelessness / Lack of optimism  Loneliness  Identity |
| Feeling Out of Control / Unable to Cope | Agitation and Anxiety  Motivation  Appearance / Self Esteem  General Mood | |  |
| **Supplementary 2.** Health-Related Quality of Life elements important to patients at clinics and support groups  *HCP= Healthcare professional*   \|  \| Symptoms & Side Effects \| \| --- \| --- \| \|  \| Mood & Emotions \| \|  \| Functionality & Day-to-Day Living \| \|  \| Relationships & Social Life \| \|  \| Patient Health Info Needs \| \|  \| Patient-Clinician Communication \| \|  \| Overall Health-Related Quality of Life \| |  |  | Planning for the Future  Housing  Accomplishments and Personal Development  Conduct & behaviour  Hobbies |
|  | Independence  Lifestyle Changes  Self-care  Travel | |  |
|  | Impact of Illness on Family  Support from Family & Friends  Family Life  Family’s Own Support | | Caring responsibilities  Social Life  Interacting with Others with Cancer |
|  | Family’s Health Information Needs  Patient Health Information Needs  Access to Test Results / Medical Records | |  |
|  | Support from HCPs  HCP collaboration  Being Able to Ask Questions | |  |
|  | General Comments on Condition  General Feelings on Diagnosis  General Comments on Symptoms & Side Effects  Feelings About Death And Dying  General Comments on Health-Related Quality of Life | |  |
